# Indomethacin enhances anti-tumor efficacy of a MUC1 peptide vaccine against breast cancer in MUC1 transgenic mice

**DOI:** 10.1371/journal.pone.0224309

**Published:** 2019-11-06

**Authors:** Jennifer M. Curry, Dahlia M. Besmer, Timothy K. Erick, Nury Steuerwald, Lopamudra Das Roy, Priyanka Grover, Shanti Rao, Sritama Nath, Jacob W. Ferrier, Robert W. Reid, Pinku Mukherjee

**Affiliations:** 1 Department of Biological Sciences, University of North Carolina at Charlotte, Charlotte, NC, United States of America; 2 Molecular Biology and Genomics Laboratory, Carolinas Medical Center, Charlotte, NC, United States of America; 3 Department of Bioinformatics and Genomics, University of North Carolina at Charlotte, Charlotte, NC, United States of America; 4 OncoTAb, Inc., Charlotte, NC, United States of America; University of Nebraska Medical Center, UNITED STATES

## Abstract

In recent years, vaccines against tumor antigens have shown potential for combating invasive cancers, including primary tumors and metastatic lesions. This is particularly pertinent for breast cancer, which is the second-leading cause of cancer-related death in women. MUC1 is a glycoprotein that is normally expressed on glandular epithelium, but is overexpressed and under-glycosylated in most human cancers, including the majority of breast cancers. This under-glycosylation exposes the MUC1 protein core on the tumor-associated form of the protein. We have previously shown that a vaccine consisting of MUC1 core peptides stimulates a tumor-specific immune response. However, this immune response is dampened by the immunosuppressive microenvironment within breast tumors. Thus, in the present study, we investigated the effectiveness of MUC1 vaccination in combination with four different drugs that inhibit different components of the COX pathway: indomethacin (COX-1 and COX-2 inhibitor), celecoxib (COX-2 inhibitor), 1-methyl tryptophan (indoleamine 2,3 dioxygenase inhibitor), and AH6809 (prostaglandin E_2_ receptor antagonist). These treatment regimens were explored for the treatment of orthotopic MUC1-expressing breast tumors in mice transgenic for human MUC1. We found that the combination of vaccine and indomethacin resulted in a significant reduction in tumor burden. Indomethacin did not increase tumor-specific immune responses over vaccine alone, but rather appeared to reduce the proliferation and increase apoptosis of tumor cells, thus rendering them susceptible to immune cell killing.

## Introduction

Breast cancer is the most common cancer diagnosed in women. In 2018, more than 266,000 women in the United States were diagnosed with invasive breast cancer, and nearly 41,000 died from complications of this disease [1]. Surgical removal is often a successful treatment for early tumors that are localized to the breast [2]. However, breast tumors have the ability to metastasize to distant sites, such as lymph nodes, lungs, liver, bones, and brain. Metastatic breast cancer is incurable, and is responsible for the majority of breast cancer deaths [3]. It is for this reason that research now focuses on the development of novel immunotherapies, including cancer-specific vaccines, for the treatment of breast cancer [4]. Vaccines are non-toxic therapies that have shown promise for the treatment of primary tumors and metastases [5–7]. Cancer vaccines are designed to immunize patients to tumor antigens, in order to stimulate the immune system to fight cancer cells while sparing normal cells [8].

Human mucin 1 (MUC1) is a transmembrane mucin glycoprotein that is expressed on the apical surface of glandular and luminal epithelial cells in many different tissues, including the breast. MUC1 contains an extracellular domain comprised of tandem repeats (TR) of 20 amino acids that are extensively O-glycosylated, a transmembrane domain, and a cytoplasmic tail (CT) [9–11]. In the vast majority (>90%) of adenocarcinomas, including most breast tumors, MUC1 is overexpressed and is distributed throughout the tumor mass and on the surface of tumor cells. In addition, tumor-associated MUC1 (tMUC1) is hypo-glycosylated, exposing the protein core [12–16]. These attributes make tMUC1 a prime target for tumor-specific immunotherapeutic strategies [17].

Our lab has previously demonstrated the effectiveness of MUC1-directed tumor vaccines in breast [12], colorectal [18], and pancreatic cancer models [19]. However, immunosuppression within the tumor microenvironment hinders the immune response to anti-cancer vaccines [20, 21]. For instance, cyclooxygenase 2 (COX-2) is an enzyme that converts arachidonic acid to prostaglandins [22]. COX-2 activity is induced in breast cancer and is involved in multiple aspects of tumorigenesis, including angiogenesis, invasion, and tumor-induced immune suppression [23–25]. COX-2 exerts its immunosuppressive effects through prostaglandin E_2_ (PGE_2_), which suppresses the functions of cytotoxic CD8^+^ T lymphocytes, T helper (T_h_) lymphocytes, natural killer (NK) cells, and dendritic cells (DCs) [26]. In breast cancer patients, COX-2 overexpression is characteristic of large, advanced tumors [27], and has been shown to reduce T cell and DC function [28].

Celecoxib, a specific COX-2 inhibitor, has been extensively used as a chemoprevention strategy for breast, colorectal, and other cancers [29–33]. In an attempt to ameliorate tumor-associated immunosuppression, our lab previously combined DC-based vaccine therapy with celecoxib treatment in a spontaneous mouse model of breast cancer [34]. In this study, we demonstrated that celecoxib increased the clinical efficacy of the vaccine. Further, COX-2 inhibition reduced breast tumor levels of indolamine 2, 3-dioxygenase (IDO) [34]. IDO is an enzyme that catabolizes L-tryptophan to L-kynurenine, and its activity is increased in breast tumors, as well as in tumor-associated antigen presenting cells (APCs) [35–38]. Depletion of tryptophan by IDO within the tumor microenvironment can lead to T-cell anergy and apoptosis [39–41]. An inhibitor of IDO, 1-methyl-tryptophan (1-MT) has shown strong anti-tumor effects in both *in vitro* and *in vivo* models of cancer [42]. Moreover, PGE_2_, the downstream product of COX-2, has been shown to regulate IDO function [43]. COX-2, PGE_2_, and IDO have also been linked with the presence of regulatory T cells (Tregs) and myeloid-derived suppressor cells (MDSCs) in the tumor microenvironment [44].

In the present study, we sought to explore the potential of combining a MUC1-specific peptide vaccine with COX pathway inhibitors for treating invasive breast cancer. Specifically, we deployed these combinational treatment regimens in transgenic mice that express human MUC1 (MUC1.Tg mice), and which were orthotopically injected with a murine syngeneic breast cancer cell line expressing human MUC1 (MTag.MUC1 cells). Once breast tumors had developed in these mice, we treated them with the MUC1 vaccine in combination with a COX-1 and COX-2 inhibitor (indomethacin), a specific COX-2 inhibitor (celecoxib), an IDO inhibitor (1-MT) and a PGE_2_ receptor antagonist (AH6809) [45]. Our results indicated that indomethacin in combination with the MUC1 vaccine resulted in a significant reduction in tumor burden. All other drugs failed to increase the effectiveness of the MUC1 vaccine at the dosages tested. Indomethacin did not enhance the anti-tumor activity of immune cells, but rather appeared to reduce the proliferation and increase the apoptosis of tumor cells.

## Materials and methods

### Mice

All mice used in this study were handled and maintained under veterinary supervision in accordance with the protocol that was approved by the University of North Carolina at Charlotte Institutional Animal Care and Use Committee (IACUC) (Protocol Number: 09–027.0 and 12–009.0). The MTag.MUC1 cell line used in animal experiments tested negative for an extended panel of pathogens by Charles River Laboratories. The PyV MT spontaneous breast cancer mice were originally provided to our lab as a gift from Dr. W.J. Muller (McGill University, Toronto, Canada) [46]. In PyV MT mice, mammary gland tumors are induced by tyrosine kinase activity associated with the polyoma virus middle T Ag (MTag), which is driven by the mouse mammary tumor virus long terminal repeat [12]. Homozygous PyV MT male mice were mated to C57BL/6 female mice to maintain heterozygous PyV MT mice. PCR was carried out as previously described to identify the MTag oncogene [12, 34]. Primer pairs for the MTag oncogene are 5′-AGTCACTGCTACTGCACCCAG-3′ (282–302 bp) and 5′-CTCTCCTCAGTTCCTCGCTCC-3′ (817–837 bp). The PCR product was analyzed by size fractionation through a 1% agarose gel.

MUC1.Tg mice were originally developed in the laboratory of Dr. Sandra J. Gendler (Mayo Clinic College of Medicine, Scottsdale, AZ) [47]. Tail clips were collected from MUC1.Tg mice when they were approximately 11 days old. Genomic DNA was isolated and used to genotype the mice by polymerase chain reaction. For MUC1.Tg the primers were 5′-CTTGCCAGCCATAGCACCAAG-3′ and 5′-CTCCACGTCGTGGACATTGATG-3′ with a 341 bp amplification product that was confirmed on 1% agarose gels.

### Cell lines and culture

MTag cell lines were generated in our lab from PyV MT mice [12]. Tumors were dissected from heterozygous female PyY MT mice at pre-determined time points. The tumors were dissociated using collagenase IV (Worthington Biochemical Corporation) and the resulting cell line was designated as MTag cells. MTag cells were maintained in complete DMEM (Invitrogen) supplemented with 10% FBS (GE Healthcare Life Sciences), 1% glutamax (Invitrogen), and 1% penicillin/streptomycin (Corning Life Sciences).

### Retroviral infection

For retroviral infection of MTag cells, GP2–293 packaging cells (stably expressing the *gag* and *pol* proteins) were co-transfected with the full-length human MUC1 construct expressing the VSV-G envelope protein. Cells were selected with 300 ug/ml G418 (Thermo Fisher Scientific), beginning 48 hours post-infection. Expression of the constructs was stable throughout the span of experiments. MUC1-positive cells were sorted using the FACSAria (BD Biosciences) to achieve 88% purity. MTag cells retrovirally infected with the full length human MUC1 plasmid are referred to as MTag.MUC1 cells.

### Drug preparations

To prepare 1-MT for oral gavage, 1 g of 1-dl-MT (MilliporeSigma) was added to a 15 ml conical tube with 7.8 ml Methocel/Tween [0.5% Tween/0.5% Methylcellulose (v/v in water) (MilliporeSigma)]. The following day, the 1-MT concentration was adjusted to 85 mg/ml by adding an additional 4 ml Methocel/Tween and mixing again briefly. For *in vitro* use, 1-MT was prepared as a 20 mM/L stock in 0.1 N NaOH, adjusted to pH 7.4 and stored at -20°C protected from light. To prepare indomethacin (MilliporeSigma), a stock solution was made at a concentration of 50 mg/ml in 100% ethanol, and heated to dissolve. Thereafter, indomethacin stock solution was diluted 1:10 in 25% Solutol. Celecoxib (MilliporeSigma) was prepared by dissolving 100 mg in 0.5 ml of DMSO for 2–3 hours at 37°C, creating a stock solution of 200 μg/μL. Stock celecoxib was diluted 1:100 in water. AH6809 (Cayman Chemical) was prepared by dissolving 1 mg into 500 μL of Solutol (heated at 60°C in order to get into solution).

### Vaccine formulation

The vaccine consisted of 100 μg each of two MHC class I-restricted MUC1 peptides, APGSTAPPA and SAPDTRPAP; 140μg of one MHC class II helper peptide TPPAYRPPNAPIL (Hepatitis B virus core antigen sequence 128–140); 100μg of mouse unmethylated CpG oligodeoxynucleotide constructs (CpG ODN); and 10,000 Units (2μg) GM-CSF (Biolegend), all emulsified in IFA [18, 19]. The vaccine was administered to the mice by intraperitoneal injection.

### *In vivo* drug treatments of tumor-bearing mice

Based on preceding titration experiments, female MUC1.Tg mice aged 8–12 weeks were orthotopically injected with 1x10^6^ MTag.MUC1 cells (in 100 μL of PBS/Matrigel) into the mammary fat pad (n = 24). On day 8 post tumor injection (p.t.i.), tumors of at least 3 mm length x 3 mm width (measured with calipers) were detected. At this point, the mice were randomly divided into 5 groups: vaccine only (n = 4), vaccine + celecoxib (n = 5), vaccine + indomethacin (n = 5), vaccine + AH6809 (n = 5), vaccine + 1-MT (n = 5). All mice were vaccinated on days 8, 19, 34, and 35 p.t.i., and were treated with celecoxib, indomethacin, or AH6809 once daily, or 1-MT twice daily, five days per week. Celecoxib was administered by oral gavage at 10 mg/kg/dose. Indomethacin was administered by oral gavage at a dose of 3 mg/kg/dose (0.1 cc/20 g mouse). 1-MT was administered by oral gavage at 400 mg/kg/dose (0.1 cc/20 g mouse). AH6809 was injected intraperitoneally at a dose of 200 μg. The mice were observed daily for signs of pain and distress, and tumor size was monitored by caliper measurements every other day until sacrifice. Body weight was measured every other day. Tumor volume was calculated according to the following formula: volume (cm^3^) = [(length in cm) x (width in cm)^2^]/2 [48]. Drug treatments were continued until the mice were sacrificed on days 34 and 35 p.t.i, which corresponds roughly to 3.5 weeks of total treatment. Mice were euthanized using CO_2_ asphyxiation followed by cervical dislocation. At this time, the mice were not yet presenting with clinical signs indicative of severe morbidity.

To conduct experiments comparing indomethacin alone, vaccine alone, and indomethacin + vaccine, 8-12-week-old female MUC1.Tg mice were orthotopically injected with 1x10^6^ MTag.MUC1 cells (in 100 μL of PBS/Matrigel) into the mammary fat pad (n = 23). On day 6 p.t.i., tumors of at least 3 mm x 3 mm were detected. At this point, the mice were randomly divided into 4 groups: untreated control (n = 6), indomethacin alone (n = 5), vaccine alone (n = 6), and indomethacin + vaccine (n = 6). During the previously described experiment, indomethacin treatment five days per week was found to cause minor dehydration in the mice. Thus, for this experiment, all indomethacin groups were gavaged three times weekly (Monday, Wednesday, Friday) with 3 mg/kg/dose. All vaccine groups were vaccinated as previously. The mice were monitored daily for signs of pain and distress. Tumor size was monitored by caliper measurements three times per week and body weight was measured twice weekly. Tumor volume was calculated according to the formula: volume (cm^3^) = [(length in cm) x (width in cm)^2^]/2. Drug treatments were continued until the mice were sacrificed on days 27 and 28 p.t.i., which corresponds roughly to 3 weeks of total treatment. Mice were euthanized using CO_2_ asphyxiation followed by cervical dislocation. At this time, the mice were not yet presenting with clinical signs that would indicate severe morbidity.

### ELISA

PGE_2_ levels in the tumor lysate from treated and control mice were determined using a specific ELISA kit for PGE_2_ metabolite (PGEM) (Cayman Chemical) from treated and control mice. All tumor lysates were made in tissue lysis buffer containing 20 mM/L HEPES, 0.15 M/L NaCl, and 1% Triton X-100 supplemented with 80 μL/ml phosphatase inhibitor cocktail II (MilliporeSigma) and 10 μL/ml complete protease inhibitor cocktail (Boehringer Mannheim GmbH). The PGEM assays were performed according to the manufacturer's recommendation. Lysates were diluted appropriately to ensure that readings were within the limits of accurate detection. Results are expressed as pg of PGEM per ml. The circulating levels of anti-MUC1 antibody response to the immunizing peptide [49] was measured using a specific ELISA. The ELISA was performed using serum (diluted 1:50) on ELISA plates coated overnight with the immunizing peptide (1 mg/ml) as the antigen. Results are expressed as absorbance.

### Histology

Immunohistochemistry (IHC) was performed on tumor sections as previously described [50]. Briefly, paraffin-embedded blocks were prepared and 4-micron thick sections were cut and mounted on slides for staining. Tissue sections were deparaffinized and hydrated via washes with xylene, 100% EtOH, 95% EtOH, 70% EtOH, and water. Antigen retrieval was performed for 40 min at 99°C followed by a 20 min cool down at room temperature in 1X Dako target antigen retrieval solution (Agilent). Tissue sections were then incubated in 2% hydrogen peroxide (diluted in 100% methanol) for 10 min. Tissue sections were blocked in 50% FBS (diluted in 1X PBS) for 45 minutes, and then incubated overnight at 4°C with 200 μl diluted TAB004-HRP (Oncotab, Inc.). The samples were then treated for 2 h at room temperature with 1/200 of anti-mouse secondary conjugated to HRP (Agilent). Negative control tumor sections were treated with the secondary only. 3,3”-Diaminobenzidine (DAB) (Vector Laboratories) was used as the chromogen and hematoxylin was used as counterstain. Slides were then dehydrated in increasing concentrations of EtOH (70%, 95%, 100%) and xylene, coverslipped with permount, and viewed under light microscopy. TUNEL assay was used to assess apoptotic cells in tumor sections using the ApopTag Peroxidase *in situ* apoptosis detection kit (MilliporeSigma). Immunoreactivity was assessed using light microscopy, and images were taken at 100X magnification. Adobe Photoshop was used to quantify the number of brown pixels per unit of viewing area (10 fields counted; n = sections from 5 mice per group).

### IFN-γ ELISPOT

At time of sacrifice, T cells from tumor draining lymph nodes (TDLNs) were isolated from tumor-bearing MUC1.Tg mice from the untreated, vaccine only, indomethacin only, or indomethacin + vaccine treatment groups, and used as responders in an IFN-**γ** ELISPOT assay. The stimulators were autologous bone marrow-derived DCs pulsed with the immunizing peptides (10 μg/ml peptide #1 APGSTAPPA; 10 μg/ml peptide #2 SAPDTRPAP)[49] for 2 h at 37°C. Following peptide stimulation, LPS (1μg/ml) (BD Biosciences) was added and incubated overnight to mature the DCs. DCs were analyzed by flow cytometry for appropriate maturation markers. The DCs were irradiated (3000 rad) using the RS 2000 irradiator (Rad Source Technologies). A responder (1x10^5^ cells/ml) to stimulator (1x10^4^ cells/ml) ratio of 10:1 was used. The responders and stimulators were incubated for 18 h on the ELISPOT plates before staining for the spots using standard IFN-γ ELISPOT plates (Mabtech). MUC1-specific spots were determined using the capture IFN-γ Ab as recommended by the manufacturer. Control wells contained T cells stimulated with either DCs that had been pulsed with irrelevant peptide (vesicular stomatitis virus peptide, RGYKYQGL), or un-pulsed DCs. Spot numbers were determined using computer-assisted video image analysis (Zellnet Consulting). Splenocytes from C57BL/6 mice stimulated with ConA (10 μg/ml)(MilliporeSigma) were used as positive control.

### Western blot

All tumor and MTag.MUC1 cell lysates were derived as previously described [51–53]. Protein concentration was determined by BCA assay (Pierce) and lysates (100 μg/lane) were resolved by SDS-PAGE on 10–15% resolving gels. Gels were blotted and probed for extracellular MUC1 (TAB004), intracellular MUC1 (CT2), COX-1, COX-2, OPG, Arginase, and CCN1. β-actin was used as the loading control. Antibodies were purchased from Santa Cruz Biotechnology.

### 3H-thymidine incorporation assay

MTag.MUC1 cells were serum-starved for 24 hr and then treated with vehicle control or 100 μM indomethacin for 24 hr. Then, effector cells isolated from the TDLNs of mice treated with the MUC1 peptide vaccine were co-cultured with MTag.MUC1 cells at 50:1 and 25:1 effector:target ratios. 3H-thymidine (PerkinElmer, Inc.) was added to the culture for 24 hr. The plates were washed to remove the lymphocytes, and incorporated 3H-thymidine was evaluated using the Topcount microscintillation counter. Counts per minute were normalized to vehicle control levels. All determinations were performed in triplicate.

### ^51^Cr release assay

MTag.MUC1 tumor cells were pretreated with 100 μM of indomethacin for 24 hr, and then labeled with ^51^Cr (PerkinElmer, Inc.). Effectors cells from the TDLNs of vaccinated mice were co-cultured with ^51^Cr-labeled tumor cells at a 100:1 ratio, and ^51^Cr release was measured after 4 hr. Spontaneous and maximal lysis was measured for MTag.MUC1 cells treated with each concentration of indomethacin. Percent lysis was calculated using the following formula: (experimental cpm—spontaneous cpm)/(maximum cpm—spontaneous cpm) x 100.

### RNA microarray

Tumor sections from untreated mice, indomethacin only mice, vaccine only mice, and vaccine + indomethacin mice were placed in RNAlater Stabilization Solution (Invitrogen) and sent to the Molecular Biology and Genomics Core Facility, Levine Cancer Center, Carolinas Medical Center. RNA was extracted from each sample, reverse transcribed, amplified and labeled using 3’ IVT Express Kit (Affymetrix Inc.). The resultant labeled complementary RNA (cRNA) was purified and fragmented as per vendor’s instructions. The cRNA samples together with probe array controls were hybridized onto Affymetrix® HT MG-430 PM array strips which cover over 39,000 mouse transcripts and variants selected from GenBank®, dbEST, and RefSeq. Hybridization controls were spiked into the cRNA samples in order to monitor and troubleshoot the hybridization process. Probes for housekeeping genes were used to assess sample integrity. Hybridization, washing, staining and scanning were performed using Affymetrix GeneAtlas® personal microarray system instruments. Affymetrix GeneAtlas® instrument control software version 1.0.5.267 was used to analyze microarray image data and to compute intensity values. Affymetrix.CEL files containing raw, probe-level signal intensities were analyzed using Partek Genomics Suite version 6.6.12.0713 (Partek). Using R/Bioconductor, robust multichip averaging (RMA) was used for background correction, quantile normalization and probe set summarization with median polish [54]. Statistical difference was calculated by two-way ANOVA analysis with a false discovery rate (FDR) set at of 0.05 and a log2 fold chance (lfc) of 0. Contrasts were set up for comparison as Vaccine vs. Vaccine & Indomethacin and Indomethacin vs. Vaccine & Indomethacin. A pairwise comparison analysis using the empirical Bayes (eBayes) method was conducted using the defined contrasts. The resulting p-values of the eBayes method were adjusted using a multiple testing adjustment method, furthermore every gene was evaluated during the multi-test to see if they fulfilled the necessary requirements to be considered differential expressed. The Benjamini-Hochberg (BH) method was used for p-value correction which was globally implemented, meaning the contrasts were considered to be independent of one another Pathway analysis was performed using Ingenuity Pathway Analysis software (APA, Ingenuity Systems, Inc.).

Contrasts were set up for comparison as Vaccine vs. Vaccine & Indomethacin and Indomethacin vs. Vaccine & Indomethacin. A pairwise comparison analysis using the empirical Bayes (eBayes) method was conducted using the defined contrasts. The resulting p-values of the eBayes method were adjusted using a multiple testing adjustment method, furthermore every gene was evaluated during the multi-test to see if they fulfilled the necessary requirements to be considered differential expressed. The Benjamini-Hochberg (BH) method was used for p-value correction which was globally implemented, meaning the contrasts were considered to be independent of one another, this was done to ensure cutoff consistency for p-values over the contrasts. A false discovery rate (FDR) of 0.05 and a log2 fold chance (lfc) of 0 were defined as the cutoffs for differential expression.

### Statistical analysis

Data were analyzed using GraphPad Prism software, with the particular statistical methods noted in the figure legend for each experiment. Results in all experiments are expressed as mean ± SEM. (*p < 0.05, **p < 0.01, ***p < 0.001, ****p < 0.0001).

## Results

### MTag.MUC1 cell line and orthotopic tumors express MUC1, COX-1, and COX-2

We sought to study the effects of a MUC1 vaccine, alone and in combination with several different COX inhibitors, on breast cancer progression in an immunocompetent mouse model. To this end, we developed a syngeneic murine breast tumor cell line that expresses the human form of MUC1. Mammary gland tumors from PyV MT mice were dissected and dissociated using collagenase IV. The cell line generated from these tumors was designated as MTag cells [55, 56]. We then transfected MTag cells with the full-length human MUC1 plasmid; the transfected cells were designated MTag.MUC1 cells.

**Fig 1:**
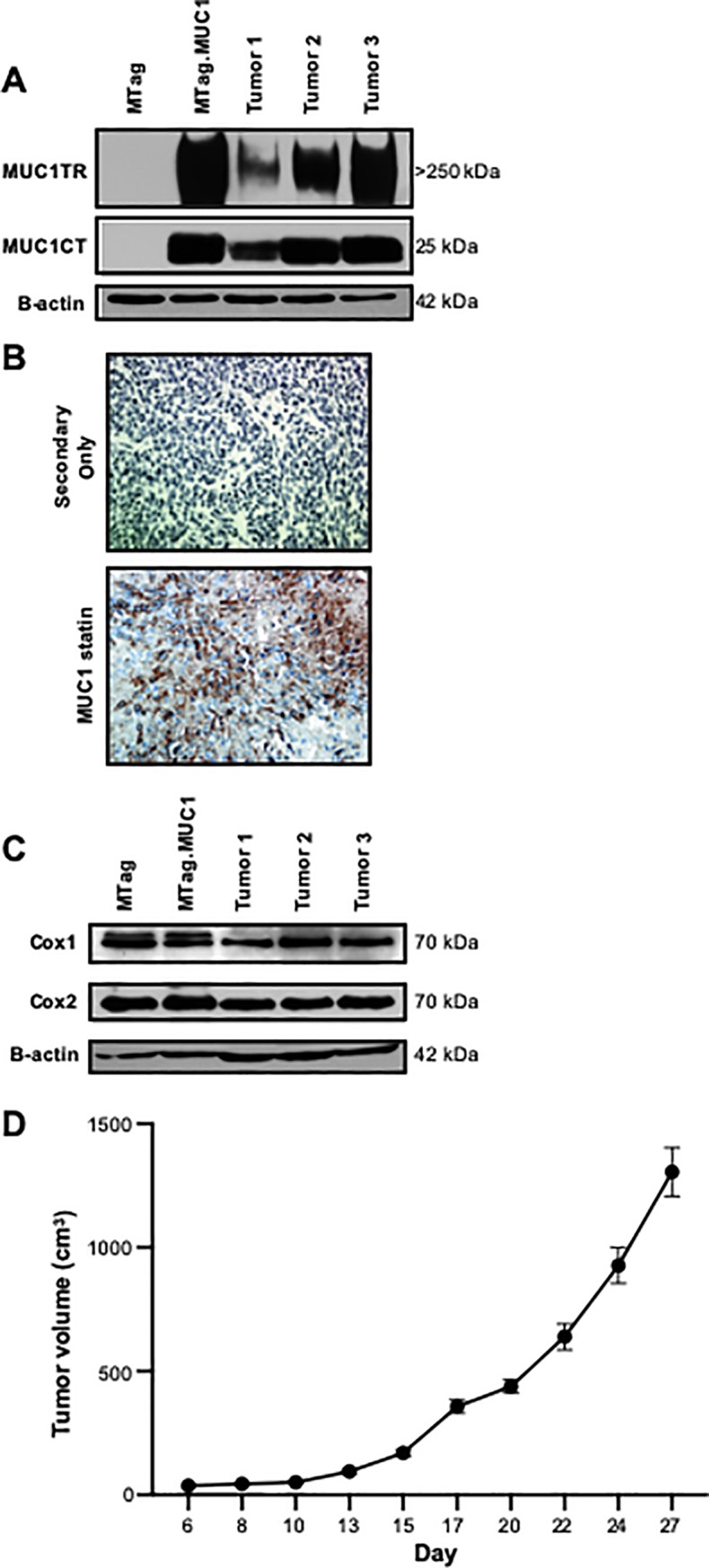
MTag.MUC1 tumors express human MUC1, COX-1, and COX-2 *in vitro* and *in vivo*. **(A)** Extracellular MUC1 (MUC1TR) and the cytoplasmic tail of MUC1 (MUC1CT) were assessed using Western blot on lysates of the MTag and MTag.MUC1 cell lines, and from orthotopic tumors removed from MUC1.Tg mice. Representative blots from 3 tumors, as well as MTag and MTag.MUC1 cells are shown. **(B)** Representative images of paraffin-embedded tumor sections that were stained with the TAB004 extracellular MUC1 antibody. **(C)** COX-1 and COX-2 protein levels were assessed using Western blot on lysates of the MTag and MTag.MUC1 cell lines, and from orthotopic tumors removed from MUC1.Tg mice. Representative blots from 3 tumors, as well as MTag and MTag.MUC1 cells are shown. **(D)** Female MUC1.Tg mice (n = 6) were injected with 1x10^6^ MTag.MUC1 cells in the mammary fat pad. Tumor volumes were determined every other day starting at day 8 p.t.i. After 27 days, the mice were euthanized and the tumors were removed.

Extracellular and intracellular levels of MUC1 were assessed in the MTag and MTag.MUC1 cells, as well as in tumors from the orthotopically injected MTag.MUC1 cells into the mammary fat pad of MUC1.Tg mice. High levels of both extracellular and intracellular MUC1 protein were detected in the lysates from the MTag.MUC1 cell line, and tumors formed in the MUC1.Tg mice but not in the MTag cells (Fig 1A). IHC staining of the MTag.MUC1 tumor sections confirmed high levels of MUC1protein expression (Fig 1B). We also assessed levels of COX-1 and COX-2 protein in the lysates of MTag and MTag.MUC1 cell lines and MTag.MUC1 tumors, and found that both COX-1 and COX-2 were expressed equally in MTag and MTag.MUC1 cells and in MTag.MUC1 tumors (Fig 1C). Lastly, to assess that MTag.MUC1 cells can form tumors, cells were injected orthotopically into the mammary fat pad of human MUC1.Tg mice, where they developed into mammary tumors (Fig 1D).

### Indomethacin significantly enhances the efficacy of MUC1 peptide vaccine therapy

**Fig 2:**
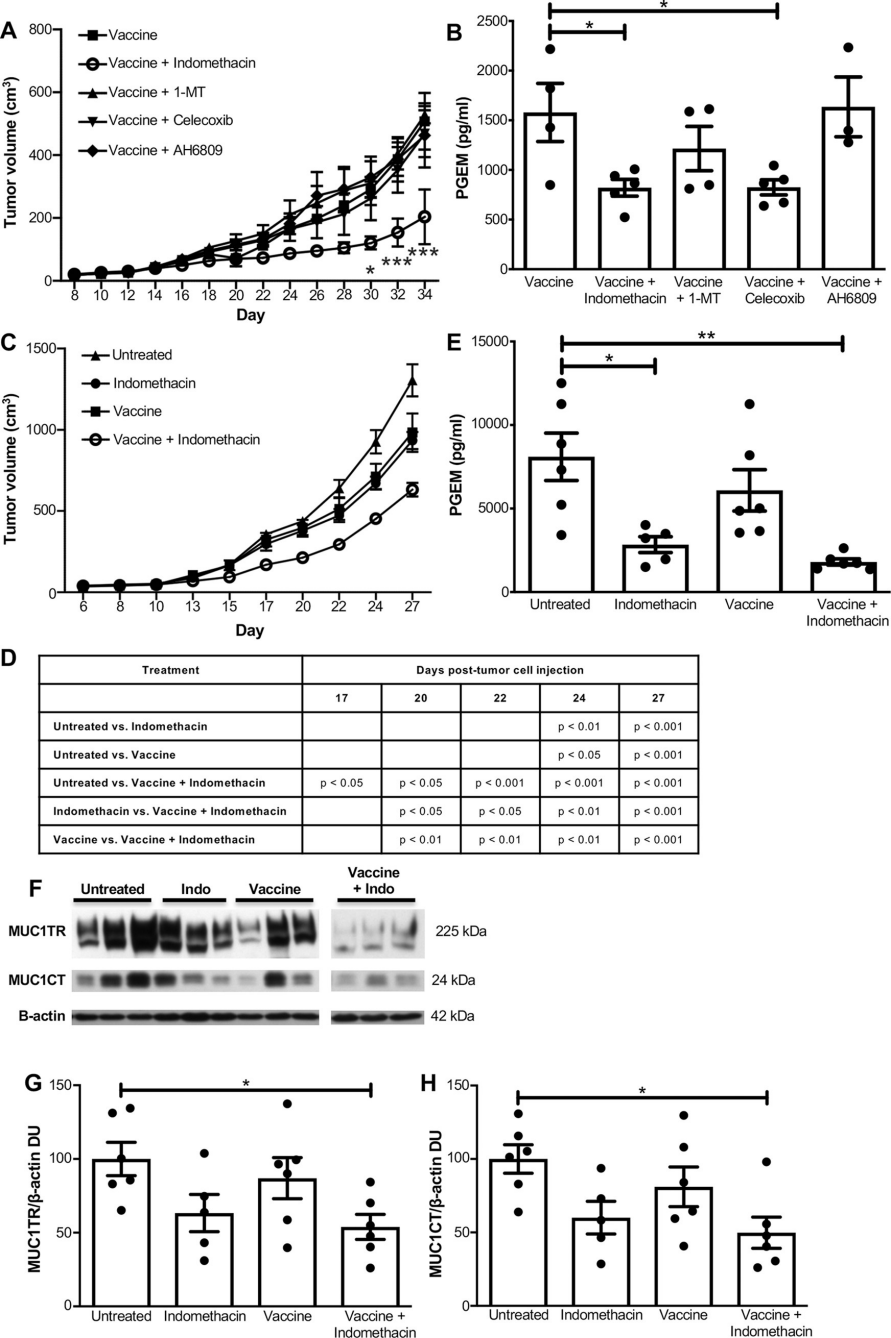
Combinational treatment of vaccine + indomethacin significantly reduces tumor growth and reduces intratumoral PGEM and MUC1 levels. Female MUC1.Tg mice with orthotopic MTag.MUC1 tumors were treated with the MUC1 peptide vaccine alone (n = 4) or in combination with indomethacin (n = 5), celecoxib (n = 5), 1-MT (n = 5), or AH6809 (n = 5). After 34 days, the mice were euthanized and the tumors were removed. (A) Tumor volumes were determined every other day starting at day 8 p.t.i., and then compared between treatment groups using a two-way ANOVA with a Bonferroni post-hoc test. Asterisks indicate significance of combinational treatment groups compared to vaccine alone. Data were generated from one experiment with 5 mice per group. (B) ELISA was used to measure PGEM in tumor lysate as a readout for PGE_2_ levels. Each combinational treatment group was compared to the MUC1 peptide vaccine alone group using a one-way ANOVA with a Dunnett’s multiple comparisons post-hoc test. Asterisks indicate significance of combinational treatment groups compared to vaccine alone. (*p < .05; ***p < 0.001). Female MUC1.Tg mice with orthotopic Mtag.MUC1 tumors were left untreated (n = 6) or treated with the MUC1 peptide vaccine alone (n = 6), indomethacin alone (n = 5), or MUC1 peptide vaccine + indomethacin (n = 6). After 27 days, the mice were euthanized and the tumors were removed. (C) Tumor volumes were determined every other day starting at day 6 p.t.i., and then compared between groups using repeated measures ANOVA with a Bonferroni post-hoc test. (D) Table showing significant differences in tumor burden between groups at various time points p.t.i. Data were generated from one experiment with the aforementioned number of mice (n = 5 or 6) per group. (E) ELISA was used to determine PGEM levels in the tumor lysates as a readout for PGE_2_ levels. Each treatment group was compared to the untreated control group using one-way ANOVA with Tukey’s post hoc test. (F) Extracellular MUC1 (MUC1TR) and the cytoplasmic tail of MUC1 (MUC1CT) were assessed in tumor lysates using Western blot. Representative blots from 3 mice per group are shown. Pixels of MUC1TR (G) and MUC1CT (H) from Western blots were normalized to β-actin, and then untreated mice were set to 100% MUC1 expression. Each treatment group was compared to the untreated control group using one-way ANOVA with Tukey’s post hoc test. Asterisks indicate significance of the comparison between the vaccine + indomethacin group and the untreated control group (*p < 0.05; **p < 0.005).

In order to investigate the effects of the MUC1 vaccine combined with the various COX pathway inhibitors, female MUC1.Tg mice with orthotopic MTag.MUC1 tumors were randomly assigned to five different treatment groups: vaccine alone, or vaccine given in combination with one of four drugs targeting the COX pathway: indomethacin, celecoxib, AH6809, or 1-MT. Of the four drugs tested, indomethacin significantly impeded tumor growth starting at day 30 p.t.i., compared to mice that received vaccine only. None of the other drug treatments–celecoxib, AH6809, or 1-MT significantly affected tumor growth (Fig 2A). To test the functionality of indomethacin and celecoxib treatment, we used ELISA to assess levels of PGEM in the tumor lysates of all treatment groups. PGEM levels were significantly reduced by both indomethacin and celecoxib treatment, indicating that these two treatments reduced COX-2 activity within the tumor microenvironment (Fig 2B).

Having identified that indomethacin improved the efficacy of the MUC1 vaccine, we next sought to determine the relative anti-tumor effects of vaccine + indomethacin compared to vaccine or indomethacin treatment alone. Female MUC1.Tg mice with MTag.MUC1 tumors were divided into four treatment groups: untreated control, vaccine alone, indomethacin alone, or vaccine + indomethacin. Indomethacin alone and MUC1 peptide vaccine alone resulted in a significantly reduced tumor burden compared to the untreated control mice starting at day 24 p.t.i. However, combining vaccine + indomethacin treatment resulted in a significant decrease in tumor growth compared to the untreated control mice starting at day 17 p.t.i. The decrease in tumor growth in the combinational therapy mice was significantly greater than that of the mice treated with indomethacin alone or vaccine alone, starting at day 20 p.t.i. (Fig 2C). Significance between groups were calculated and tabulated in Fig 2D for days 17, 20, 22, 24, and 27 post tumor challenge.

Intratumoral PGEM levels were significantly reduced in both mice treated with indomethacin alone, and those treated with indomethacin combined with the MUC1 vaccine (Fig 2E). Since MUC1 expression is associated with tumor aggressiveness, we assessed intratumoral level of MUC1 expression from all treatment groups. Compared to untreated control mice, we observed that combinational treatment resulted in a significant decrease in intratumoral MUC1TR and MUC1CT levels by western blotting (Fig 2F–2H). Together, these data indicated that combinational therapy was more effective at delaying MUC1^+^ tumor growth than either single therapy arm.

### MUC1 peptide vaccine and combinational therapy elicit anti-MUC1-specific IFN-γ and antibody responses *in vivo*

**Fig 3:**
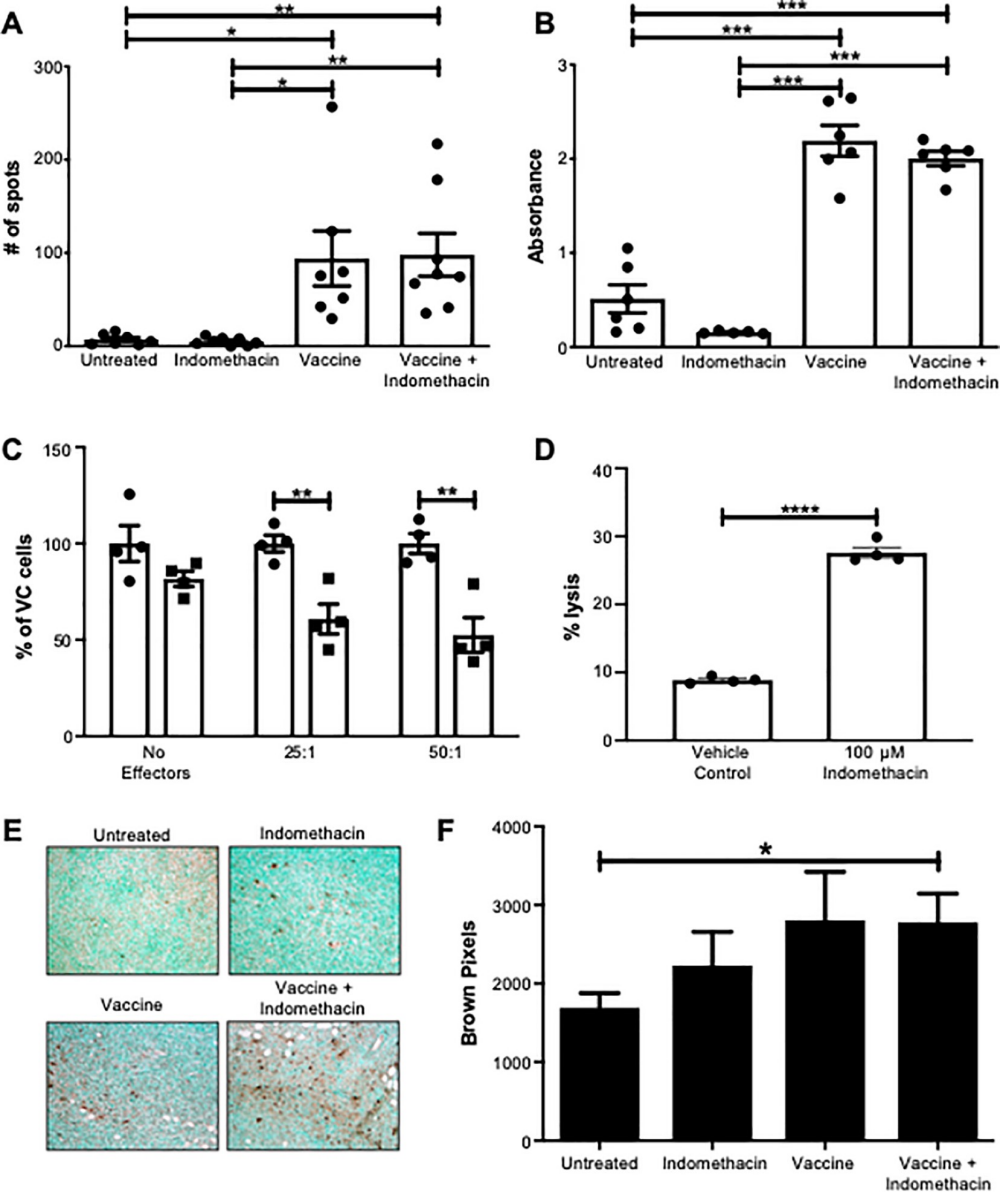
**A and B. Treatment with MUC1 peptide vaccine induces anti-MUC1 immune responses *in vivo*.** Female MUC1.Tg mice with orthotopic Mtag.MUC1 tumors were left untreated (n = 6) or treated with the MUC1 peptide vaccine alone (n = 6), indomethacin alone (n = 5), or MUC1 peptide vaccine + indomethacin (n = 6). After 27 days, the mice were euthanized and the tumors and TDLNs were removed. Data were generated from one experiment with the aforementioned number of mice (n = 5 or 6) per group. **(A)** ELISPOT was used to assess IFN-γ production by T cells in the TDLNs in untreated mice, as well as mice treated with the MUC1 vaccine alone, indomethacin alone, or vaccine + indomethacin. **(B)** ELISA was used to assess the endogenous antibody response to the immunizing peptide in sera (diluted 1:50) from untreated mice, as well as mice treated with the MUC1 vaccine alone, indomethacin alone, or vaccine + indomethacin. **C-F. MUC1 peptide vaccine results in reduced proliferation and increased apoptosis of mammary tumor cells.** Female MUC1.Tg mice with orthotopic Mtag.MUC1 tumors were left untreated (n = 6) or treated with the MUC1 peptide vaccine alone (n = 6), indomethacin alone (n = 5), or MUC1 peptide vaccine + indomethacin (n = 6). After 27 days, the mice were euthanized and the tumors were removed. Data were generated from one experiment with the aforementioned number of mice (n = 5 or 6) per group. **(C)** MTag.MUC1 cells were treated with vehicle control or 100 μM indomethacin for 24 hr. Then, effector cells isolated from the TDLNs of tumor-bearing mice treated with the MUC1 peptide vaccine were co-cultured with indomethacin-treated MTag.MUC1 cells (squares) or vehicle-treated cells (circles) at 50:1 and 25:1 effector:target ratios. Tumor cell proliferation was assessed via 3H-thymidine incorporation, and counts per minute were normalized to vehicle control (VC) treated cells. Comparisons between groups were conducted using a two-way ANOVA with a Bonferroni post-hoc test. **(D)** MTag.MUC1 tumor cells were pretreated with 100 μM indomethacin for 24 hr and then labeled with ^51^Cr. Effector cells from the TDLNs of vaccinated mice were co-cultured with ^51^Cr-labeled tumor cells at 100:1 ratio, and ^51^Cr release was measured after 4 hr. Comparison between groups was conducted using an unpaired Student’s T-test. **(E-F)** Apoptotic cells within the tumors were assessed via TUNEL stain, and the number of brown pixels per unit viewing area was quantified using Adobe Photoshop (10 fields per slide were counted). Unpaired Student’s T-test was used for pairwise comparisons between all groups of mice (*p < 0.05, **p < 0.01, ***p < 0.001).

Next, we wanted to determine if indomethacin improves the efficacy of the MUC1 vaccine by increasing the immune response to MUC1^+^ tumor cells. To this end, we used ELISPOT to assess IFN-γ production in response to the MUC1 vaccine in tumor-draining lymph nodes (TDLNs) of tumor-bearing mice from the untreated, vaccine alone, indomethacin alone, and indomethacin + vaccine mice. We observed a significant increase in the number of spots, indicating high levels of MUC1-specific IFN-γ-producing T cells, in both vaccinated and combinational therapy mice. However, the difference in the level of spots between vaccinated and combinational therapy mice was negligible (Fig 3A). A similar pattern was observed in the levels of circulating antibody produced against the immunizing peptide of MUC1 [49], as both vaccine and combinational therapy resulted in significantly increased antibody levels over untreated mice and mice treated with indomethacin alone. However, no difference was observed between vaccine and combinational therapy mice (Fig 3B). These results indicated that indomethacin treatment does not significantly improve the anti-MUC1 specific immune responses as measured by IFN-γ-ELISOPT or antibody production over vaccine treatment alone.

### Indomethacin treatment curbs proliferation of tumor cells and renders cells more susceptible to immune cell killing

Since indomethacin treatment did not enhance the anti-tumor activity of MUC1 vaccine, we hypothesized instead that indomethacin acts on tumor cells to render them more sensitive to immune-mediated killing. To assess any effects on the proliferation of tumor cells, we vaccinated tumor-bearing mice with the MUC1 peptide vaccine, and isolated effector lymphocytes from the TDLNs. Separately, we treated MTag.MUC1 cells with increasing doses of a vehicle control (VC) or indomethacin *in vitro*. After 24 hr, the media was discarded, and the cells were washed to remove any remaining indomethacin. Then, effector lymphocytes isolated from TDNLs of vaccine-treated tumor-bearing mice were added to the Indomethacin treated or untreated Mtag.MUC1 cells at 50:1 and 25:1 effector:target ratios. The ability of the effector lymphocytes to impede tumor cell proliferation was assessed by the addition of ^3^H-thymidine for 24 hr. The plates were washed to remove the lymphocytes, and ^3^H-thymidine incorporation into the MTag.MUC1 tumor cells was evaluated. The effector cells significantly impeded proliferation of MTag.MUC1 cells that were pre-treated with 100 μM of indomethacin compared to cells that were pre-treated with vehicle control. (Fig 3C).

We also performed a chromium release assay using the same effector lymphocytes isolated from vaccinated tumor-bearing MUC1.Tg mice. The target MTag.MUC1 tumor cells were pretreated with 100 μM indomethacin for 24 hr, and then labeled with ^51^Cr and plated at effector:target ratios of 100:1. We were able to observe effective tumor cell killing only when the MTag.MUC1 tumor cells had been pre-treated with 100 μM indomethacin (Fig 3D). These results demonstrated that indomethacin treatment renders Mtag.MUC1 tumor cells more vulnerable to immune-mediated killing.

To determine if these *in vitro* effects were occurring *in vivo*, we investigated the effects of indomethacin on tumor cell proliferation and apoptosis in tumor sections from tumor-bearing mice that has been left untreated, treated with vaccine or indomethacin alone, or vaccine + indomethacin. We observed no difference in PCNA protein levels between the treatment groups (S1A and S1B Fig). However, TUNEL staining showed a trend of increased apoptosis in the indomethacin and vaccine treated groups compared to control. However, only the combinational treatment resulted in a significant increase in TUNEL^+^ cells compared to the untreated control (Fig 3E and 3F). These results suggest that combinational treatment with indomethacin and the MUC1 peptide vaccine resulted in a significant increase in apoptosis of breast cancer cells *in vivo*.

### Upregulation of *Arg1* and *Chi3l3* and downregulation of *Tnfrsf11b* gene expression in vaccine + indomethacin treated tumors

**Fig 4:**
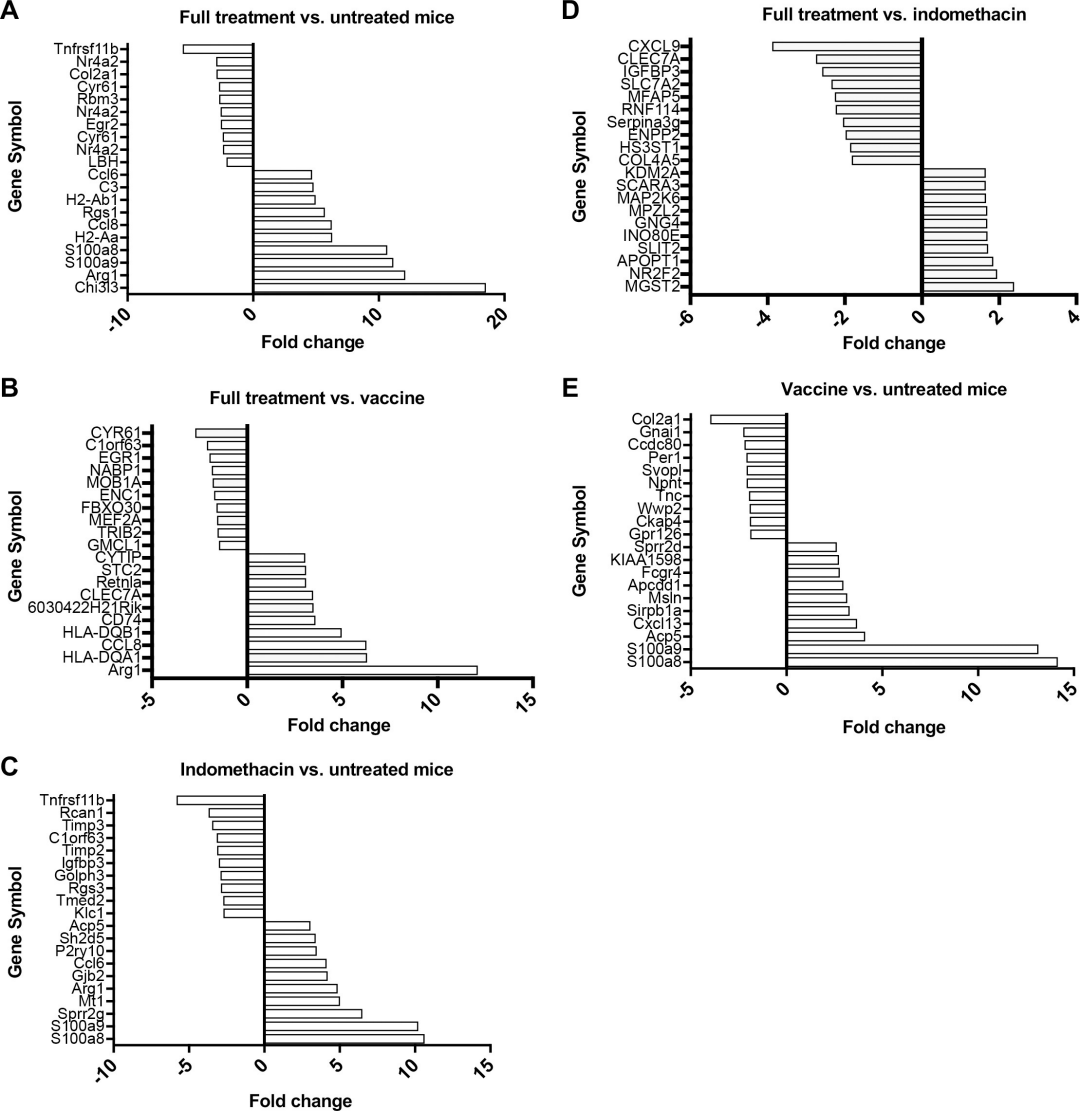
Microarray analysis of tumors from mice treated with MUC1 peptide vaccine, indomethacin, or vaccine + indomethacin. RNA was isolated from the orthotopic tumors of MUC1.Tg mice that were left untreated, as well as mice that were treated with the MUC1 peptide vaccine, indomethacin, or vaccine + indomethacin. Microarray analysis was conducted, and the top ten up-regulated and down-regulated genes from **(A)** the full treatment vs. untreated control, **(B)** full treatment vs. vaccine alone, **(C)** indomethacin vs. untreated control, **(D)** full treatment vs. indomethacin alone, and **(E)** vaccine vs. untreated control are shown. Comparison between groups was conducted using a two-way ANOVA with false discovery rate (FDR) (n = 2 per group).

We next analyzed gene expression to further investigate potential mechanisms for the increased tumor cell killing seen with indomethacin + vaccine treatment. To this end, we conducted microarray analyses of RNA isolated from the orthotopic tumors of MUC1.Tg mice from the different treatment groups. The top ten up-regulated and down-regulated genes were compared between treatment groups (Fig 4). Interestingly, several genes related to immune system function, such as *CCL6* [57], *CCL8* [58], MHC class II molecules, and arginase-1 (*Arg1*) [59] were highly up-regulated in the vaccine + indomethacin treatment group compared to untreated control (Fig 4A). *Arg1* expression was also up-regulated when we compared vaccine + indomethacin treatment to vaccine alone (Fig 4B) or indomethacin to untreated control (Fig 4C). In contrast, *Arg1* expression was not one of the ten most up-regulated genes in the full treatment vs. indomethacin (Fig 4D) or vaccine vs. untreated control (Fig 4E). These findings suggested that indomethacin treatment was responsible for *Arg1* up-regulation. Indomethacin treatment also decreased the expression of several genes involved in immune system regulation and cancer progression. In both the full treatment vs. untreated control (Fig 4A) and indomethacin vs. untreated control (Fig 4C), the top down-regulated gene was *Tnfrsf11b*, which encodes osteoprotogerin (OPG), a decoy receptor for RANKL [60] and TNF-related apoptosis-inducing ligand (TRAIL) [61]. Additionally, *Cyr61* (CCN1), which promotes breast tumor angiogenesis [62, 63], was down-regulated in the combinational treatment group compared to both untreated controls (Fig 4A) and vaccine alone (Fig 4B). It is also clear that the MUC1 vaccine by itself significantly upregulates S100a8 and S100a9, both involved in formation of inflammatory protein complex from neutrophils with broad apoptosis inducing activity. This remains upregulated in the vaccine + indomethacin group.

Additional analysis of the RNA microarray data revealed that *Igfbp3* expression was upregulated in the combination treatment group compared to all other groups (S1 Table). The *Igfbp3* gene encodes the protein insulin-like growth factor-binding protein 3 (IGFBP3), a prominent binding partner of insulin-like growth factors (IGF) I and II. IGPBP3 is also known to act independently of IGF to inhibit cell division and promote apoptosis [64].

### Upregulation of Arg1 and downregulation of OPG protein expression in the vaccine + indomethacin treated tumor confirms gene expression data

**Fig 5:**
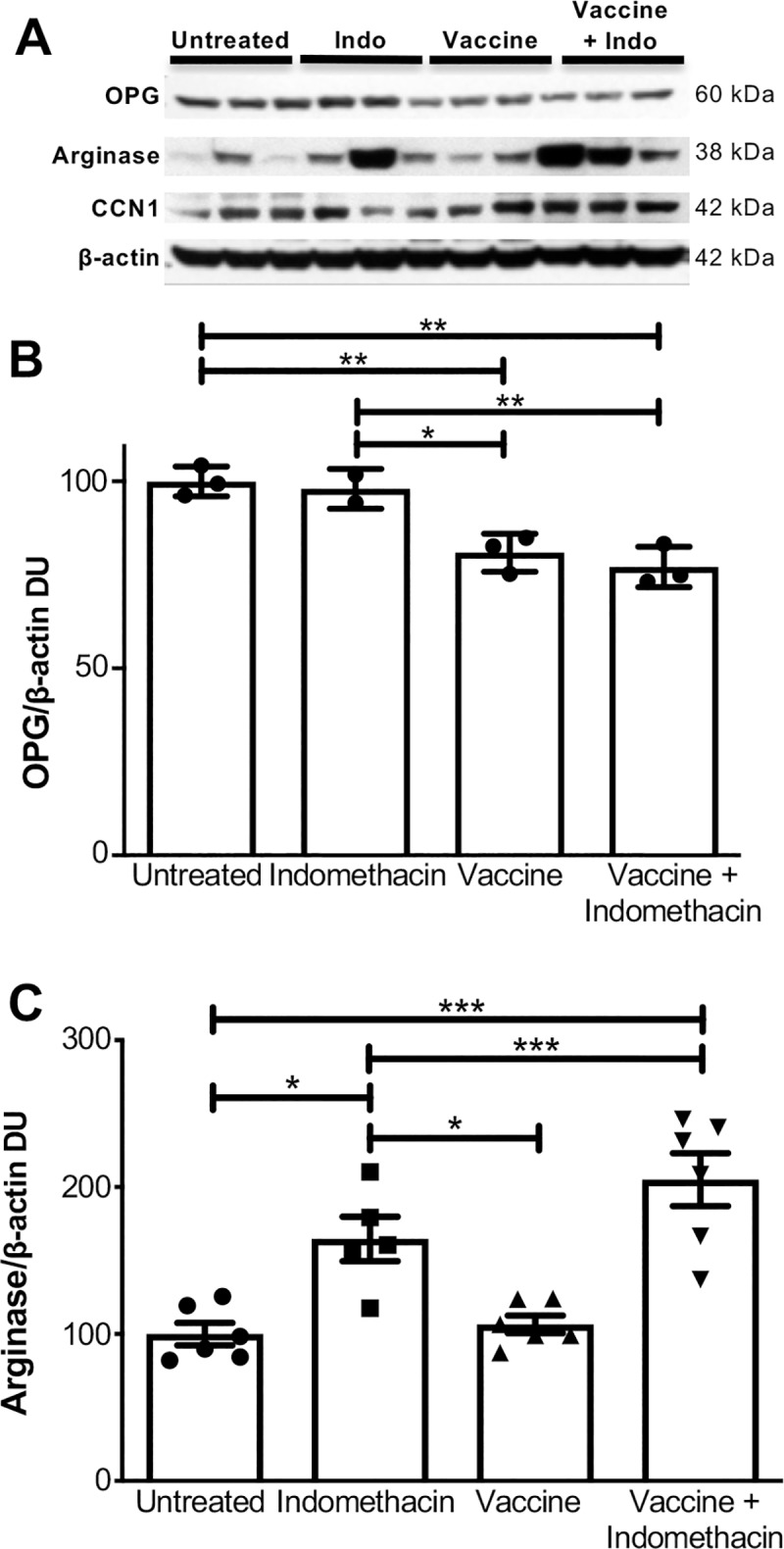
Combinational therapy with indomethacin and MUC1 peptide vaccine significantly reduces osteoprotegerin (OPG) and significantly increases arginase 1 levels within the tumors of mice. Female MUC1.Tg mice with orthotopic Mtag.MUC1 tumors were left untreated (n = 6) or treated with the MUC1 peptide vaccine alone (n = 6), indomethacin alone (n = 5), or MUC1 peptide vaccine + indomethacin (n = 6). After 27 days, the mice were euthanized and the tumors were removed. Data were generated from one experiment with the aforementioned number of mice (n = 5 or 6) per group. **(A)** Western blot analysis of OPG, arginase1, and CCN1 protein levels in tumor lysates from untreated control mice, as well as mice treated with indomethacin, MUC1 peptide vaccine, or indomethacin + MUC1 peptide vaccine. Representative blots from 3 mice per group (2 for vaccine + indomethacin) are shown. **(B-C)** Quantification of OPG and arginase protein signal from comparison of groups was done by one-way ANOVA with Tukey’s post hoc test (*p < 0.05, **p < 0.01, ***p < 0.001).

We conducted Western blot analysis of tumor lysates to determine if the RNA microarray findings translated to changes in protein expression. Although *Cyr61* was downregulated in the full treatment versus vaccine at the gene level, there was no significant correlation with the protein expression level of its encoded protein CCN1 (Fig 5A). Contrarily, OPG protein levels encoded by the *Tnfrsf11b* gene were significantly downregulated in full treatment and vaccine treated mice compared to untreated controls (Fig 5A and 5B) confirming the gene array data. Similarly, we observed increased ARG1 levels in the indomethacin alone and full treatment mice compared to untreated and vaccine treated tumors (Fig 5A and 5C). Altogether, these findings indicated that the MUC1 vaccine, indomethacin, and indomethacin + MUC1 vaccine treatments cause complex changes in gene expression and protein expression within the tumor microenvironment. Some of these changes point to potential mechanisms of neutrophils and macrophage activation and polarization within the tumor causing increased apoptosis in the vaccine + indomethacin group and possibly renders tumor cells more vulnerable to immune-mediated killing.

## Discussion

Within the past several years, cancer vaccines have shown promise for the treatment of many different cancers [8]. Vaccines based on human MUC1 have demonstrated particular efficacy in preclinical mouse models of breast cancer. In one recent study, a vaccine based on a MUC1 glycopeptide epitope conjugated to Tetanus Toxoid showed potent activity as a preventative vaccine against breast cancer [65]. Another study demonstrated that co-administration of a MUC1 peptide vaccine with a mutant isoform of VEGF165b produced an enhanced immune response against breast cancer [66]. Less encouragingly, results from clinical trials of MUC1-based vaccines have been mixed. Vaccination of a group of stage II breast cancer patients with oxidized mannan-MUC1 resulted in significantly lower rates of recurrence and a longer time to recurrence compared to patients that had received placebo [67, 68]. In contrast, treatment of metastatic breast cancer patients with a sialyl-Tn keyhole limpet hemocyanin (STn-KHL) vaccine produced no difference in time to progression or overall survival compared to a KHL vaccine [69]. The findings from these clinical trials suggest that MUC1-based vaccines should be tailored to particular subtypes and stages of breast cancer.

To investigate novel cancer immunotherapies, our lab has made use of the oncogenic mice that carry the polyoma virus middle T antigen driven by the MMTV promoter (PyV MT mice). These mice develop spontaneous breast tumors that metastasize to the lungs and bone marrow [12]. Within the tumor microenvironment, the immunosuppressive activities of COX-2 and its downstream products greatly reduce the effectiveness of cancer vaccines [21, 28]. We previously demonstrated that celecoxib, a specific COX-2 inhibitor, significantly augmented the effectiveness of a DC-based breast cancer vaccine in reducing primary tumor burden, preventing metastasis, and increasing survival. In that study, we found that tumor-associated COX-2 activity *in vivo* regulates IDO expression within the tumor microenvironment. Celecoxib treatment resulted in lower levels of tumor-associated IDO, which helped to improve anti-tumor cytotoxic T lymphocyte activity [34].

**Fig 6:**
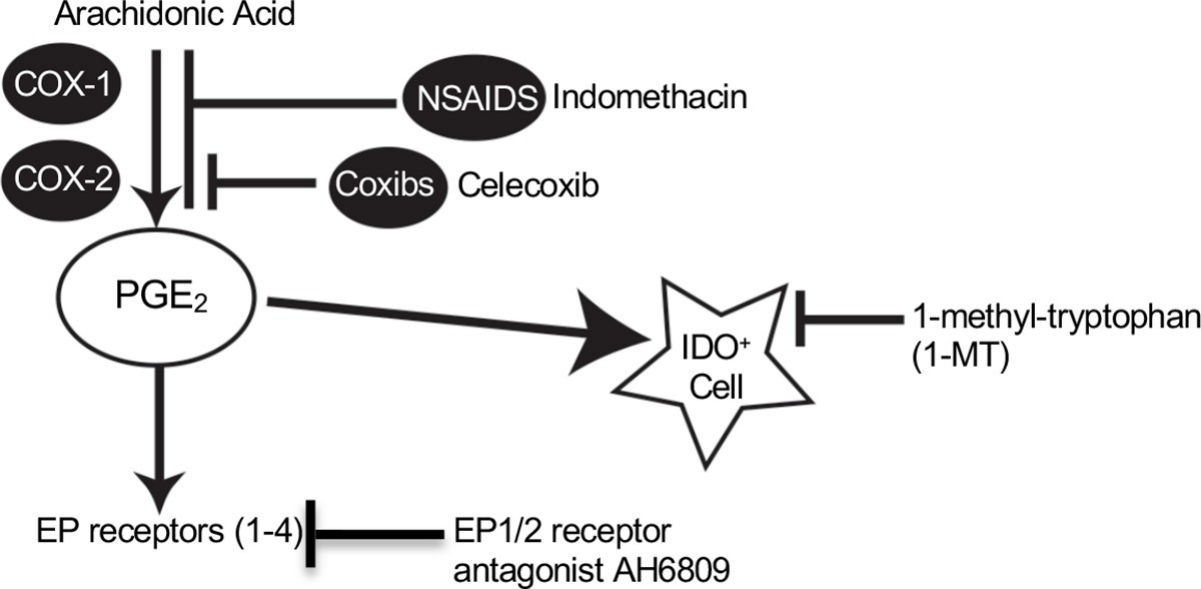
Schematic overview of the COX pathway targets of indomethacin, celecoxib, 1-MT, and AH6809.

In the present study, we generated a breast cancer cell line from the tumors of PyV MT mice, and overexpressed full length human MUC1. Using these Mtag.MUC1 cells, MUC1-expressing breast tumors were generated in the immune competent MUC1.Tg mice, which carry the human MUC1 transgene driven by its own promoter [47]. We used a MUC1 peptide vaccine that consists of two MHC class-I restricted peptides, an MHC class-II restricted helper peptide, as well as CpG-ODN and GM-CSF [18, 19]. This peptide vaccine was tested in combination with four different drugs: the COX-2 inhibitor celecoxib; indomethacin, a non-selective COX-1 and COX-2 inhibitor; 1-MT, an IDO inhibitor; and the PGE_2_ receptor antagonist AH6809. Each of these drugs interferes with different components of the COX pathway (Fig 6).

Results from this study demonstrated that combining indomethacin with the MUC1 peptide vaccine results in a significant decrease in tumor burden compared to either single treatment arm. However, indomethacin treatment did not result in increased T or B cell activity over vaccine treatment alone. Rather, our results indicated that indomethacin treatment reduces the proliferation and increases lytic activity of the effector T cells, thus increasing TUNEL positive apoptotic tumor cells *in situ* (Fig 3). To further explore the mechanisms behind this, we assessed the effects of our treatment regimens on gene expression and protein expression in the tumors. Combinational and indomethacin treatment resulted in a significant decrease in *TnfrsfIIb*/OPG expression, both at the RNA and protein level. In addition to its role in regulating RANK-RANKL binding [60], OPG also acts as a decoy receptor for TNF-related apoptosis-inducing ligand (TRAIL) [61]. TRAIL is expressed on subsets of T cells and natural killer (NK) cells [70], and has been shown to induce the apoptosis of certain types of cancer cells [71–73]. By binding TRAIL, OPG acts as an anti-apoptotic factor that can contribute to the survival of cancer cells [61, 74, 75]. Thus, reduced production of OPG could potentially facilitate increased apoptosis of tumor cells. Further studies will be required to explore any potential mechanistic links between decreased OPG expression and increased tumor cell killing.

We also observed that combinational and indomethacin treatment resulted in increased expression of arginase1, both at the RNA and protein level. Arginase metabolizes L-arginine to L-ornithine and urea [76]. Depletion of L-arginine in the tumor microenvironment is known to suppress T cell immune responses, which has emerged as a fundamental mechanism of immune evasion by cancer cells [59, 77, 78]. Thus, increased arginase expression is paradoxical to the increased effectiveness of our combinational therapy. Nevertheless, Arginase is also associated with macrophage polarization and M2 macrophages. It is possible that the combination of increased S100a and b along with increased arginase results in increased susceptibility of the tumor to T cell killing. These findings highlight the complex biological effects that can occur with combinational therapies. Some of these changes in gene and protein expression most likely promote cancer cell proliferation and tumor growth, while others inhibit these processes. The increased efficacy of the indomethacin + vaccine combinational therapy indicates that the magnitude of the anti-cancer effects is favored in this particular balance.

The RNA microarray results also revealed that indomethacin treatment induces differential expression of several additional genes involved in immune regulation and cancer progression (Fig 5B). For instance, we observed increased expression of the *HLA-DQA1* and *HLA-DQB1* MHC class II genes. Several alleles of these particular MHC class II genes have been associated with breast cancer protection and increased breast cancer risk [79, 80]. We also observed increased expression of *CCL8*, a chemokine that modulates the migration of immune cells and breast cancer cells [81]. CD74 expression was also upregulated. Expression of CD74 on tumor cells has been associated with higher MHC class II expression and a stronger intratumoral immune response in basal-like invasive breast cancer [82]. Furthermore, we observed upregulation of *CLEC7A*, which encodes the pattern recognition receptor Dectin-1 that is expressed on dendritic cells and other antigen-presenting cells [83]. Dectin-1 has been shown to be involved in the anti-tumor response of natural killer cells and CD8^+^ T cells [84]. Concurrently, we noted decreased expression of *CYR61*, an angiogenic factor that promotes breast cancer tumorigenesis and metastasis [85, 86]. However, CCN1 which is encoded by CTR61 was not significantly altered in the protein expression data (Fig 5).

In addition, we observed increased expression of *Igfbp3* in the combination treatment group. Several studies have shown that high tumor levels of IGFBP-3 are associated with more aggressive breast cancer and decreased overall survival [87–89]. A detailed analysis of IGFBP-3 expression within breast tumors uncovered that IGFBP-3 is highly expressed in stromal tissue within the tumor, but its expression is suppressed within the malignant epithelial cells. This is in contrast to healthy breast tissue, where IGFBP-3 is expressed in the stroma and epithelial cells. In light of these findings, increased IGFBP-3 expression in breast cancer could represent a mechanism by which the surrounding stromal cells are attempting to induce apoptosis of tumors cells, which have found a way to evade this mechanism [90]. Future studies should investigate the protein levels of these genes, as well as their potential mechanistic effects on tumor cell proliferation and immune-mediated killing.

In conclusion, our findings indicate that indomethacin enhances the efficacy of the MUC1 peptide vaccine in treating MUC1-expressing mammary tumors. Since celecoxib did not elicit a similar effect, the results were likely independent of COX-2 inhibition. Several studies have indicated that indomethacin and other NSAIDs exert chemopreventive effects through COX-independent means [91, 92]. For example, indomethacin has been shown to promote the apoptosis of colon cancer cells by inhibiting peroxisome proliferator-activated receptor δ (PPARδ) activity [93–95]. This is not surprising since one of the binders and activators of PPARδ is arachidonic acid. Interestingly, we noted a 1.6 fold decrease in the HDAC9 gene, one of the ligands of PPARδ, in the indomethacin treated tumors compared to the untreated tumors (data not included). Although we do not have the exact molecular mechanism deciphered, our preclinical studies offer an opportunity to assess the feasibility of inhibition of the COX pathway in combination with immunotherapy for the treatment of breast cancer. This is especially relevant at a time when long-term use of COX-2 inhibitors is under debate and safer alternative agents are desired [96].

## Supporting information

S1 Fig **(A)** Western blot analysis of PCNA protein levels in tumor lysates from untreated control mice (n = 6), as well as mice treated with indomethacin (n = 5), MUC1 peptide vaccine (n = 5), or indomethacin + MUC1 peptide vaccine (n = 6). Representative blots from 4 mice per group for untreated and vaccine + indomethacin, and 3 mice per group for vaccine only and indomethacin only are shown. **(B)** Quantification of PCNA protein signal from comparison of groups was done by one-way ANOVA with Tukey’s post hoc test.
(TIF)

S1 Table Normalized *Igfbp3* gene expression values based on RMA analysis. (TIF)
