# Supplementary figures and images for: Indomethacin enhances anti-tumor efficacy of a MUC1 peptide vaccine against breast cancer in MUC1 transgenic mice
Source: PLoS One. 2019 Nov 6;14(11):e0224309. doi: 10.1371/journal.pone.0224309 (PMC6834267; doi:10.1371/journal.pone.0224309)

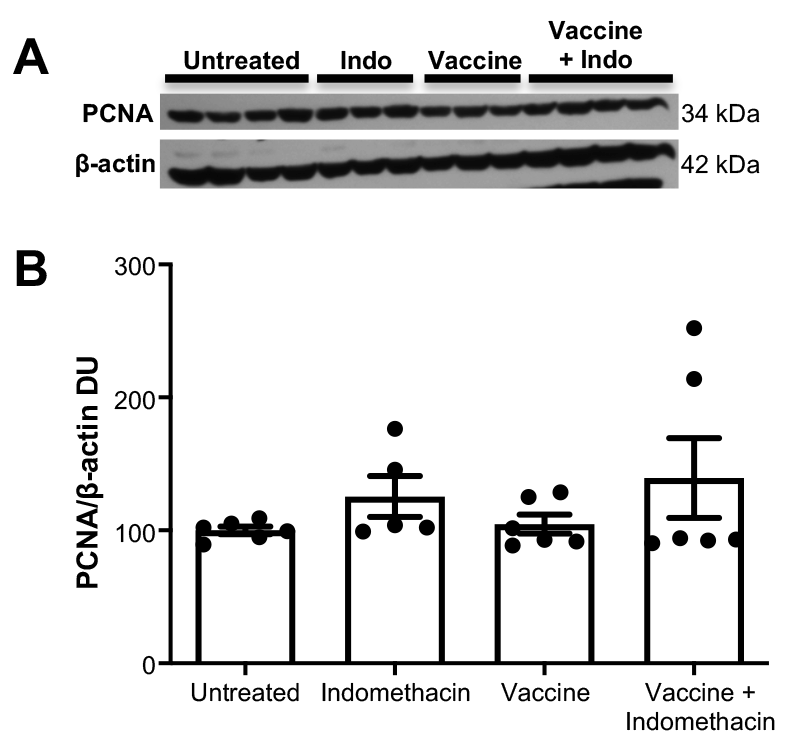

Supplement: S1 Fig — (A) Western blot analysis of PCNA protein levels in tumor lysates from untreated control mice (n = 6), as well as mice treated with indomethacin (n = 5), MUC1 peptide vaccine (n = 5), or indomethacin + MUC1 peptide vaccine (n = 6). Representative blots from 4 mice per group for untreated and vaccine + indomethacin, and 3 mice per group for vaccine only and indomethacin only are shown. (B) Quantification of PCNA protein signal from comparison of groups was done by one-way ANOVA with Tukey’s post hoc test. (TIF) [file pone.0224309.s001.tif]

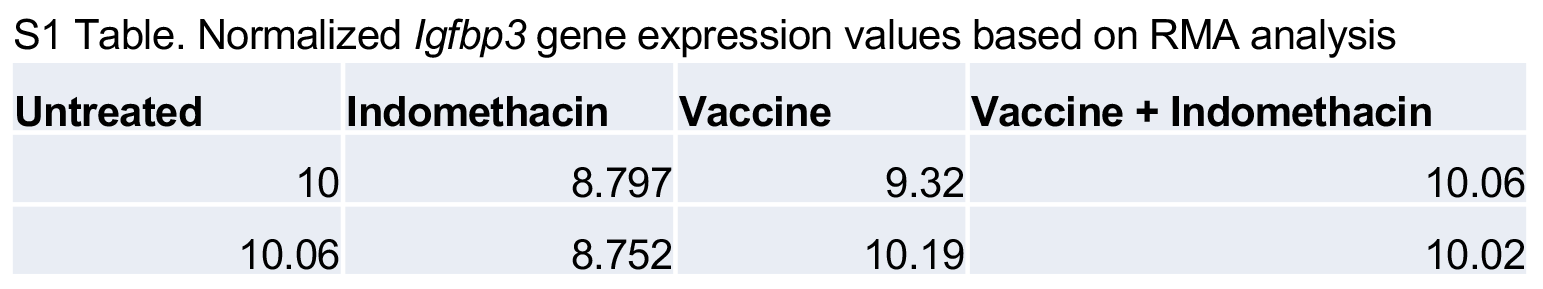

Supplement: S1 Table — (TIF) [file pone.0224309.s002.tif]
